# Transmissible gastroenteritis virus infection decreases arginine uptake by downregulating CAT-1 expression

**DOI:** 10.1186/s13567-018-0591-1

**Published:** 2018-09-20

**Authors:** Lu Xia, Lei Dai, Qian Yang

**Affiliations:** MOE Joint International Research Laboratory of Animal Health and Food Safety, College of Veterinary Medicine, Nanjing Agricultural University, Weigang 1, Nanjing, Jiangsu 210095 China

## Abstract

Transmissible gastroenteritis virus (TGEV) is a coronavirus that causes severe diarrhea in suckling piglets. TGEV primarily targets and infects porcine intestinal epithelial cells, which play an important role in nutrient absorption. However, the effects of TGEV infection on nutrient absorption in swine have not yet been investigated. In this study, we evaluated the impact of TGEV infection on arginine uptake using the porcine small intestinal epithelial cell line IPEC-J2 as a model system. High performance liquid chromatography (HPLC) analyses showed that TGEV infection leads to reduced arginine uptake at 48 hours post-infection (hpi). Expression of cationic amino acid transporter 1 (CAT-1) was attenuated as well. TGEV infection induced activation of phospho-protein kinase C α (p-PKC α), phospho-epidermal growth factor receptor (p-EGFR), and enhanced the expression of caveolin-1, all of which appear to be involved in down-regulating arginine uptake and CAT-1 expression. These results illuminate the relationship between TGEV infection and nutrient absorption, and further our understanding of the mechanisms of TGEV infection.

## Introduction

Transmissible gastroenteritis (TGE) is a highly contagious enteric disease of pigs caused by TGE virus (TGEV), with mortality rates as high as 100% in piglets less than 2 weeks old [1, 2]. TGEV infects the epithelial cells of the intestinal tract, resulting in villus atrophy and impaired absorption of nutrition [3, 4]. The porcine small intestinal epithelial cell line IPEC-J2 is an intestinal columnar epithelial cell line that was isolated from the mid-jejunum of a neonatal piglet [5]. IPEC-J2 cells are used for in vitro investigations of swine viruses and nutrition [6, 7].

Arginine, an amino acid that is essential in neonates, is synthesized by enterocytes [8, 9]. Arginine is one of the most metabolically versatile amino acids, and serves as a precursor for synthesis of protein, nitric oxide, creatine, polyamines, agmatine, and urea, which mediate important regulatory functions that affect nutrient metabolism and immune responses [10–13]. The cellular uptake of arginine is mediated primarily by proteins in the cationic amino acid transporter (CAT) family [14]. Abundant evidence indicates that this family is important for maintaining arginine homeostasis and overall protein nutrition in the body [15–17].

The molecular mechanism underlying CAT-1 expression is associated with the activation of the intracellular signal transduction molecule protein kinase C (PKC). PKC is located in close proximity to CAT-1 in the caveola and regulates CAT-1 activity [18, 19]. PKC has been identified as a family of protein kinase enzymes that participate in many cellular processes, and classical isoforms of PKC are involved in the regulation of the CAT-1 transporter [20, 21]. The role of PKC-α in the regulation of arginine transport has been investigated in different cell types [22, 23].

Intestinal arginine transport is regulated by local as well as systemic factors such as growth factors, differentiation states, and luminal substrates [24]. Several growth factors, including epidermal growth factor (EGF) and transforming growth factor α (TGF-α), elicit their functions by binding to EGF receptors (EGFR) and instigating an intracellular PKC signal transduction cascade [25, 26]. EGFR is a transmembrane protein and is expressed on the surface of many different cell types. Previous studies in our laboratory have shown that EGFR promotes TGEV entry into intestinal epithelial cells by regulating cofilin activity, and that both clathrin- and caveolin-mediated endocytosis are important for TGEV and EGFR internalization [27, 28].

Most studies have focused on the pathogenesis of TGEV infection. However, little is known about the impact of TGEV infection on nutrient uptake from the small intestine, especially for amino acids. We therefore evaluated TGEV infection on arginine uptake and the signaling pathways involved in arginine uptake in IPEC-J2 cells.

## Materials and methods

### Cell lines

IPEC-J2 cells were purchased from the DSMZ (Germany), and HEK293T cells were purchased from the American Type Culture Collection (ATCC). Both cell lines were cultured in Dulbecco’s modified Eagle’s medium (DMEM, Gibco) with 10% fetal bovine serum (FBS, Gibco), using an incubator maintained at 37 °C in 5% CO_2_.

### Viral infection

TGEV strain SHXB (GenBank: KP202848.1) was provided by the Jiangsu Academy of Agricultural Sciences (JAAS, Jiangsu Province, China) [29]. For experimental assays, cells were infected with TGEV at a multiplicity of infection (MOI) of 3 for 1 h at 37 °C in serum-free medium and washed with phosphate-buffered saline (PBS, pH 7.2) three times to remove unbound virus. Cells were then cultured in maintenance medium (DMEM containing 2% FBS).

### RNA extraction and real-time PCR analysis

Total RNA was extracted from TGEV-infected IPEC-J2 cells using TRIzol (Invitrogen), according to the manufacturer’s instructions. cDNA was synthesized by using HiScript Q RT SuperMix for qPCR (Vazyme, China), according to manufacturer’s instructions. Gene expression was measured via quantitative RT-PCR using a TaKaRa SYBR Green qPCR Kit (TaKaRa, Japan). Primers are shown in Table 1. Quantitative real-time PCR was performed with an Applied Biosystems 7500 real-time PCR System. Data were normalized against β-actin levels and are expressed as fold differences between control and TGEV-infected cells according to the 2^−∆∆CT^ method [30].

**Table 1:** **Primers used for RT-PCR**

Gene	Sequence (5′–3′)	Size (bp)
β-Actin	F: AGATCAAGATCATCGCGCCT	171
	R: ATGCAACTAACAGTCCGCCT	
CAT-1	F: AGACGGGCTGCTGTTTAAGT	131
	R: ACCGTTAAAATACCGGCGTG	
CAT-2	F: TGGATGGCACTTGGTTTCCTG	91
	R: GCAGGTGAAAGGCCTCGTAT	

### Western blotting

Protein was obtained from IPEC-J2 cells at the indicated time points post-infection using ice-cold radioimmunoprecipitation assay (RIPA) lysis buffer containing 10 mM phenylmethylsulfonyl fluoride (PMSF). Total protein concentrations were determined using a bicinchoninic acid (BCA) protein assay kit (Thermo Scientific, USA). Cell lysates that contained equal amounts of protein were denatured, subjected to 10% sodium dodecyl sulfate polyacrylamide gel electrophoresis (SDS-PAGE), and transferred to polyvinylidene difluoride (PVDF) membranes (Millipore, USA). Membranes were blocked with Tris-buffered saline (TBS) containing 5% nonfat dry milk for 2 h, and incubated with the indicated primary antibodies at 4 °C overnight. The following antibodies were used in this study: phospho-EGF receptor rabbit mAb (CST, USA); anti-CAT1 (Abcam, UK); caveolin-1 antibody, phospho-PKC α antibody, and β-tubulin antibody (EnoGene Biotech, USA). The next day, membranes were washed three times with TBST and incubated with HRP-conjugated secondary antibodies for 2 h at room temperature. The membranes were then washed with TBST for 5 min four times. Protein band detection was performed using ECL reagents (Thermo Scientific, USA).

### Lentivirus-mediated RNA interference (RNAi) depletion experiments

pLVX-shRNA is an HIV-1-based lentiviral expression vector designed to express a small hairpin RNA (shRNA) for RNA interference (RNAi) studies (Clontech, USA). The target sequences for shRNA were designed from the following sequences: NM_214007 (porcine EGFR), NM_001012613.1 (porcine CAT-1), and NM_214438 (porcine caveolin-1). HEK293T cells were transfected with 1 μg of specific expression plasmid per 10^6^ cells using the X-tremeGENE HP DNA Transfection Reagent (Roche, Switzerland), diluted in Opti-MEM (Invitrogen) in T-25 cell culture flasks. Lentiviral particles (MOI = 1) were subsequently added to IPEC-J2 cells and gently mixed.

### Arginine detection in cell culture medium using HPLC [31]

To measure arginine uptake by IPEC-J2 cells, 100 μL of medium was deproteinated with 200 μL of trichloroacetate using HPLC. The medium was vortexed and centrifuged at 13 000 rpm for 15 min at 4 °C. Supernatant was recovered using a pipette and purified by passage through a 0.22 μm filter. The derivatization procedure was a modification of the method described by Elkin et al. [32]. Next 50 μL of the derivatizing reagent acetonitrile-TEA-PITC (8:1:1:1 [v/v]) was added, and the reaction was incubated at room temperature for 60 min. A mixture of ddH_2_O–Hexane (1 mL and 0.5 mL) was added and incubated for 10 min. Finally the solvents were removed under a nitrogen stream, and the tube was sealed and stored at 4 °C pending analysis. Chromatography was carried out using a gradient elution at a constant temperature of 40 °C. Eluant A was an aqueous buffer prepared by adding 0.1 mol/L sodium acetate solution; Eluant B was acetonitrile. The gradient program is shown in Table 2. Arginine uptake was calculated as follows: arginine uptake (mg/L per 10^6^ cells) = (arginine concentration of the control group − arginine concentration of the experimental group)/cell number.

**Table 2:** **Gradient program employed for the separation of PTC-amino acids**

Time (min)	0	10	20	21	34	35	43	44	49	50	53
A% (v/v)	95	91	89	79	77	75	68	20	10	95	95
B% (v/v)	5	9	11	21	23	25	32	80	90	5	5

### Statistical analysis

All results are expressed as the means ± standard deviations (SD) from three independent experiments. Significant differences between control and experimental groups were analyzed using Student’s *t*-test. Difference were considered significant at *0.01 < *p* < 0.05, **p < 0.01.

## Results

### TGEV infection decreases arginine uptake

**Figure 1:**
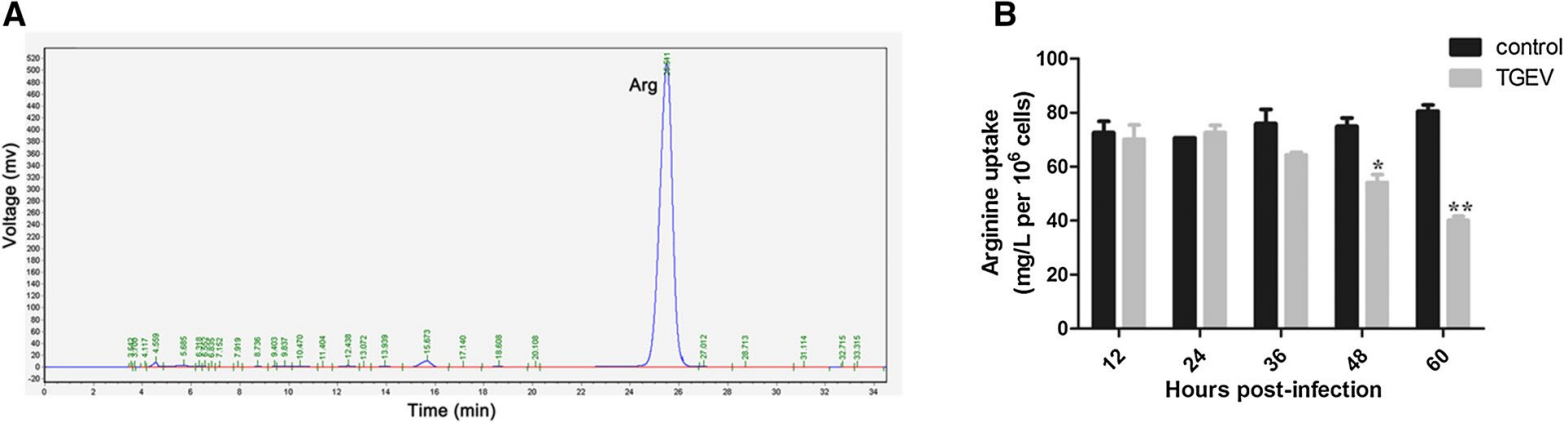
**TGEV infection decreases arginine uptake. A** HPLC analysis of a purified sample of arginine used as a standard. **B** IPEC-J2 cells were incubated with TGEV (MOI = 3), and culture supernatants were collected for arginine analysis at the indicated time points. Results are expressed as means ± SDs of three independent experiments. Differences were considered significant at *0.01 < *p* < 0.05, **p < 0.01.

The uptake of arginine was quantified by HPLC after calibrating the assay using arginine standards (Figure 1A) and preparing a standard curve (data not shown). TGEV infection had no effect on the uptake of arginine at 12 h and 24 h. Arginine uptake began to decrease at 36 h, and was significantly lower at 48 h and 60 h (Figure 1B).

### TGEV infection reduces CAT-1 expression

**Figure 2:**
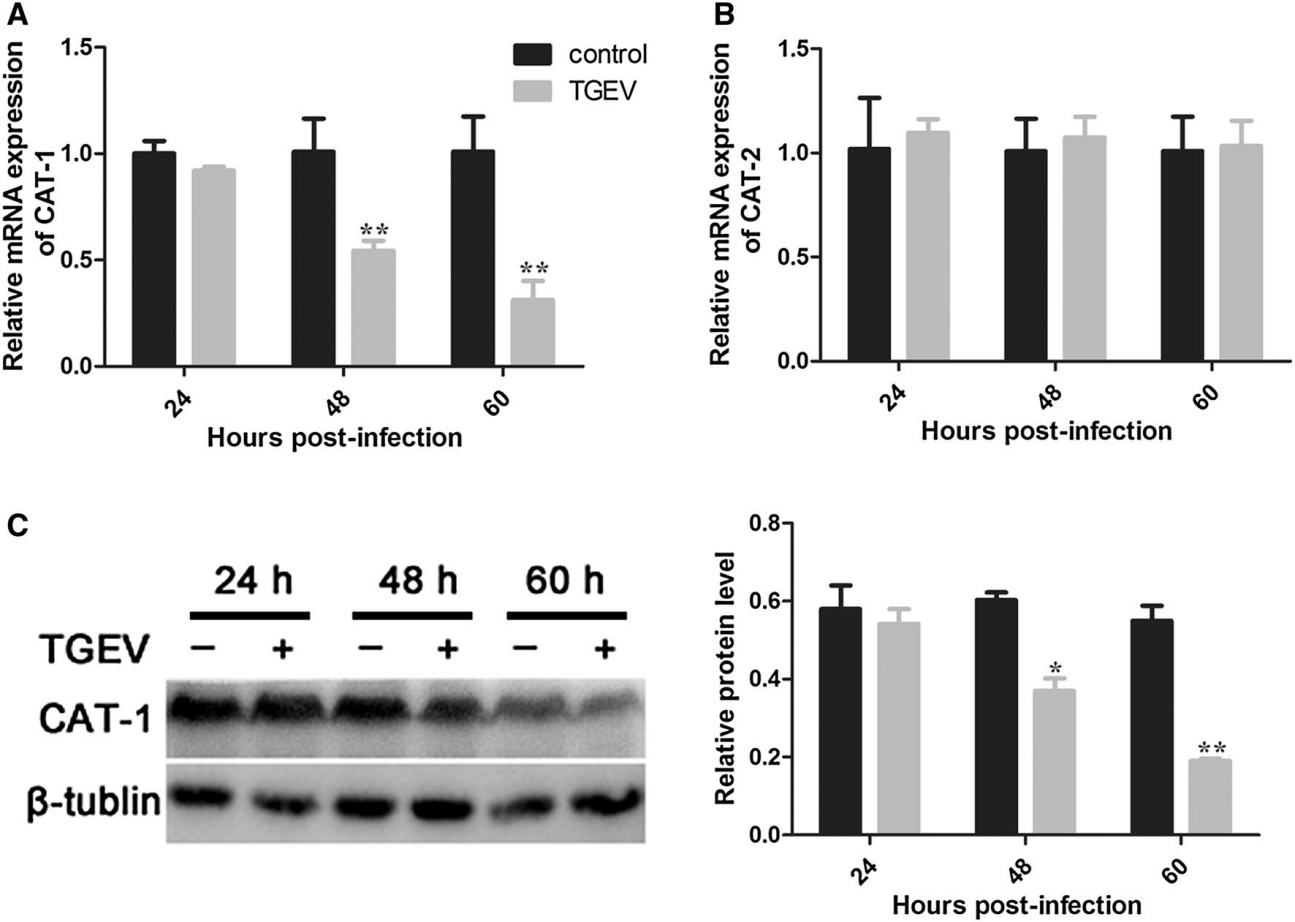
**TGEV infection decreases expression of CAT-1. A**, **B** Relative mRNA expression for CAT-1 and CAT-2, determined using RT-PCR. **C** Western blot analysis of CAT-1 protein levels. Differences were considered significant at *0.01 < *p* < 0.05, **p < 0.01.

Arginine uptake is mediated by the CAT family of transporters. mRNA levels of CAT-1 and CAT-2 were quantified by RT-PCR. Expression of CAT-1 markedly decreased at 48 h and 60 h, while mRNA levels of CAT-2 did not change significantly (Figures 2A and B). Expression of CAT-1 protein was consistent with CAT-1 mRNA expression (Figure 2C).

### PKC α is involved in arginine uptake

**Figure 3:**
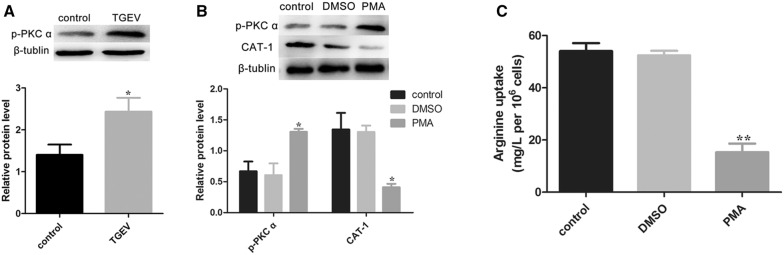
**TGEV infection activates p-PKC α and PKC α influences arginine uptake. A** Protein levels of p-PKC α were analyzed by Western blot at 48 hpi. **B** Cells pre-infected with TGEV were treated with PBS, DMSO (100 nM), or PMA (100 nM) for 12 h. Expression of p-PKC α and CAT-1 was analyzed. **C** Arginine uptake was assayed. Differences were considered significant at *0.01 < *p* < 0.05, **p < 0.01.

PKC α is involved in the regulation of CAT-1 transport activity [33]. Our results showed the activation of p-PKC α after TGEV infection at 48 h (Figure 3A). To further investigate whether PKC α was responsible for CAT-1 expression and arginine uptake, we used phorbol 12-myristate 13-acetate (PMA), a classical activator of PKC α. TGEV-infected IPEC-J2 cells that were treated with PMA for 12 h exhibited significant activation of p-PKC α, and also a noticeable decrease of CAT-1 (Figure 3B). The uptake of arginine was also dramatically reduced after treatment with PMA (Figure 3C).

### EGFR regulates CAT-1 and arginine uptake

**Figure 4:**
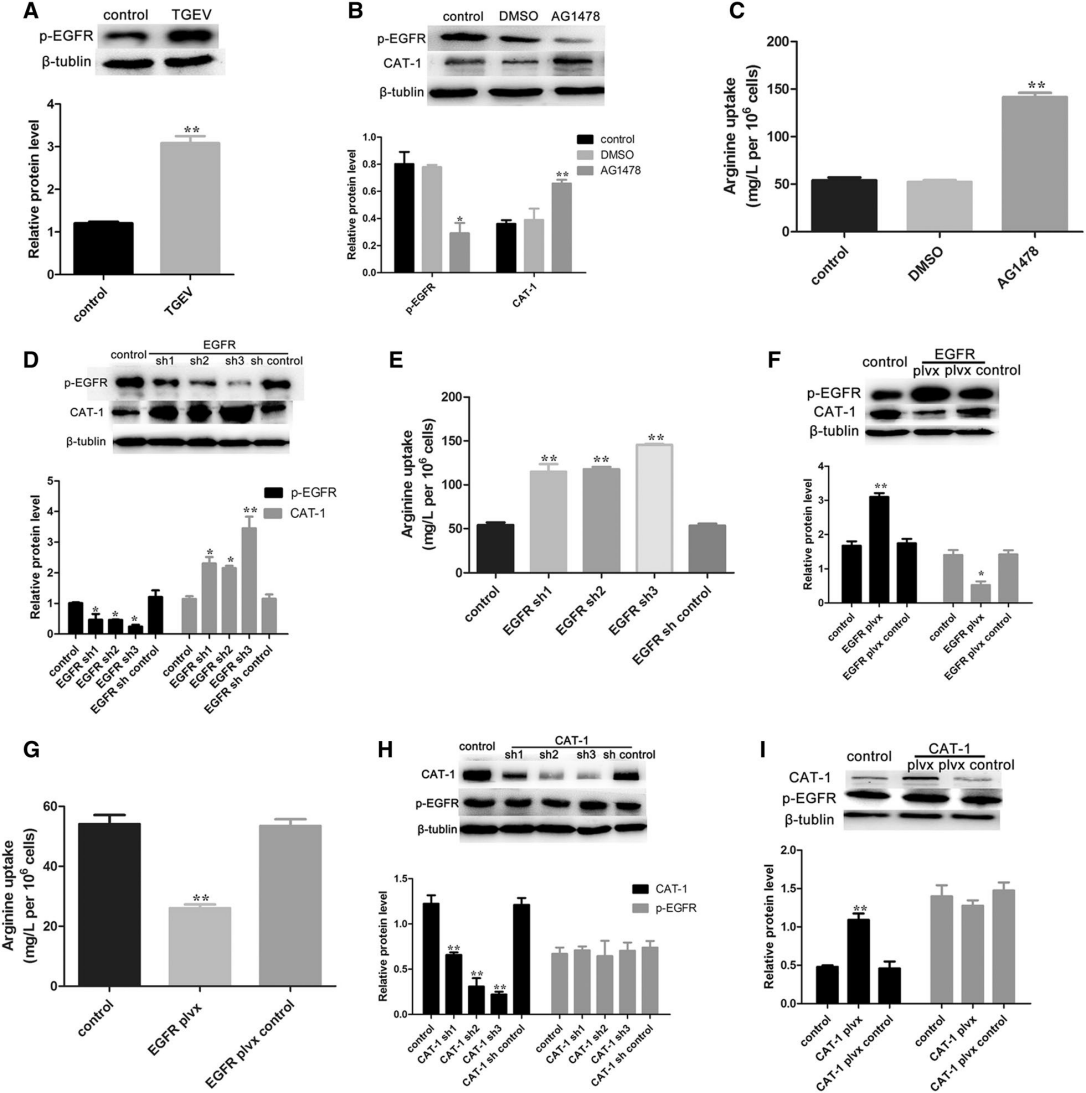
**TGEV infection decreases arginine uptake via activated p-EGFR. A** Western blot analysis was performed to determine levels of p-EGFR at 48 hpi. **B** TGEV-infected cells were treated with PBS, DMSO (100 nM), or AG1478 (100 nM) for 12 h, and then were analyzed to determine protein expression levels of p-EGFR and CAT-1. **C** Arginine concentrations in the culture medium from each group were assayed. **D** Cells were transfected with EGFR specific shRNAs or the shRNA control for 48 h. p-EGFR and CAT-1 expression were evaluated by Western blot analysis. **E** Culture medium from each group was assayed to determine arginine uptake. **F** Cells were transfected with a plvx-EGFR shRNA or the plvx control, and analyzed for p-EGFR and CAT-1 protein levels by Western blot. **G** The uptake of arginine was assayed. **H** Cells were transfected with three CAT-1 specific shRNAs or the shRNA control, and analyzed for CAT-1 and p-EGFR protein levels by Western blot. **I** Cells were transfected with a plvx-CAT-1 shRNA or the plvx control for 48 h, and analyzed for CAT-1 and p-EGFR protein expression by Western blot. Differences were considered significant at *0.01 < *p* < 0.05, **p < 0.01.

PKC acts as the downstream target of EGFR signaling, and can be regulated by EGFR [34, 35]. We therefore analyzed the expression level of p-EGFR. As expected, TGEV infection markedly induced activation of p-EGFR (Figure 4A). AG1478, an inhibitor of EGFR, was used to determine whether EGFR was involved in the process. As shown in Figure 4B, AG1478 showed a decrease of p-EGFR, while CAT-1 expression was promoted. Moreover, arginine uptake was increased after TGEV-infected cells were treated with AG1478 (Figure 4C).

To study the relationship between EGFR and CAT-1, we manipulated EGFR and CAT-1 expression in knockdown and overexpression experiments. EGFR-targeting shRNAs significantly inhibited p-EGFR expression, but expression of CAT-1 increased (Figure 4D). Arginine uptake also increased after transfecting with EGFR-targeting shRNAs (Figure 4E). In contrast, cells transfected with plvx-EGFR had higher p-EGFR expression, lower CAT-1 expression, and decreased arginine uptake (Figures 4F and G). TGEV-infected cells transfected with CAT-1 specific shRNAs exhibited reduced CAT-1 expression but no effects on EGFR (Figure 4H). Transfection with plvx-CAT-1 resulted in higher levels of CAT-1 but left EGFR unchanged (Figure 4I). Taken together, these results indicated that TGEV infection activates p-EGFR, which modulates CAT-1 and thereby affects arginine uptake.

### Caveolin-1 participates in arginine uptake

**Figure 5:**
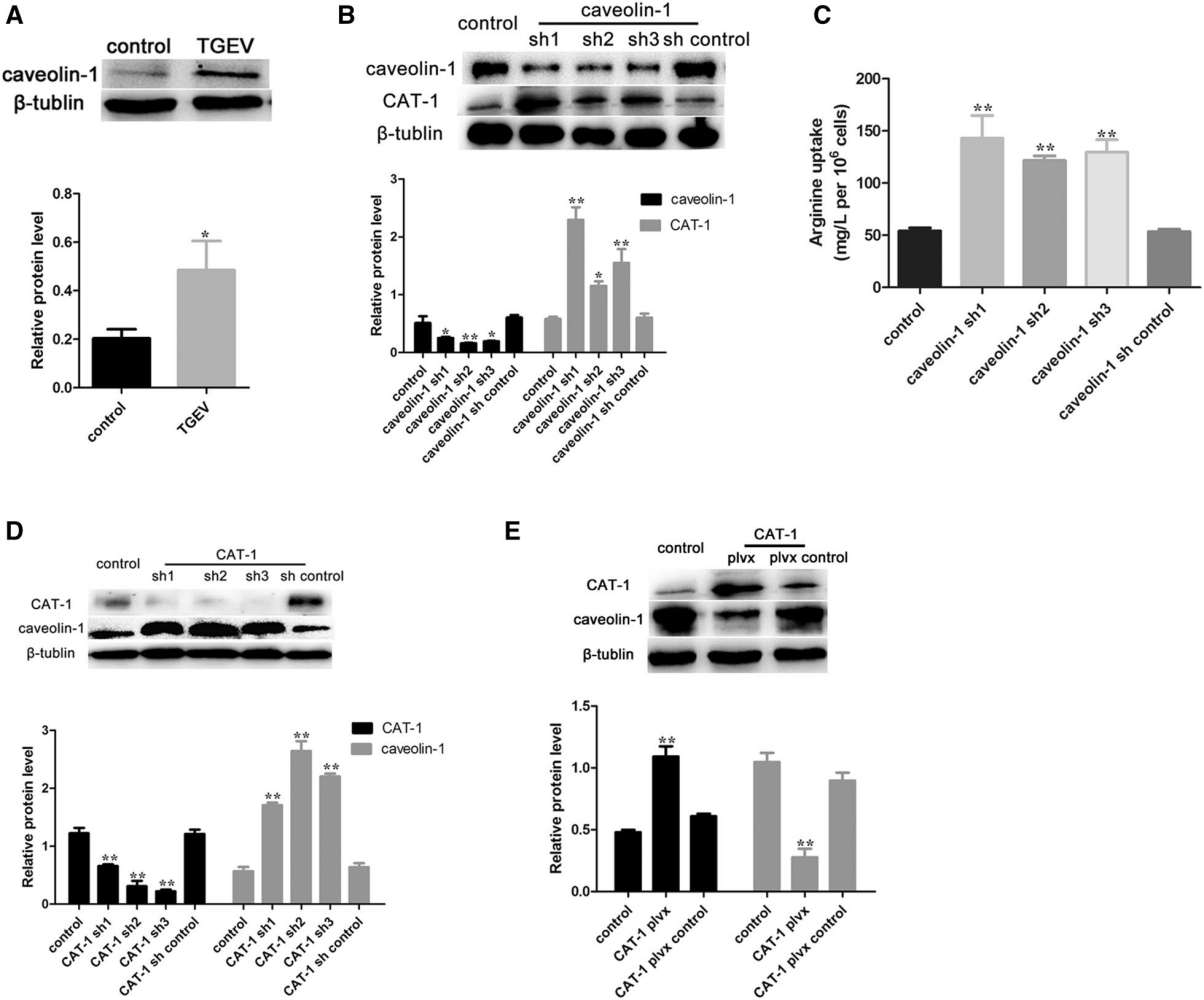
**Caveolin-1 is involved in arginine uptake during TGEV infection. A** Western blot analysis was performed to determine the levels of caveolin-1 protein 48 h after TGEV infection. **B** Cells were transfected with three caveolin-1 specific shRNAs or the shRNA control. Cell extracts were then subjected to Western blot analysis to measure expression of caveolin-1 and CAT-1. **C** The uptake of arginine was assayed. **D** Cells were transfected with three CAT-1 specific shRNAs or the shRNA control. Western blot analysis was used to measure expression of CAT-1 and caveolin-1. **E** Cells were transfected with a plvx-CAT-1 shRNA or the plvx control. Western blot analysis was used to measure expression of CAT-1 and caveolin-1 after TGEV infection. Differences were considered significant at *0.01 < *p* < 0.05, **p < 0.01.

Figure 5A shows that TGEV infection increased caveolin-1 expression. Caveolin-1 knockout suppressed the expression of caveolin-1, while increasing CAT-1 protein expression and arginine uptake (Figures 5B and C). Transfection with CAT-1-targeting shRNAs resulted in higher caveolin-1 protein expression than in control cells (Figure 5D), and plvx-CAT-1 had the opposite effect (Figure 5E).

### A signaling cascade regulates arginine uptake during TGEV infection

**Figure 6:**
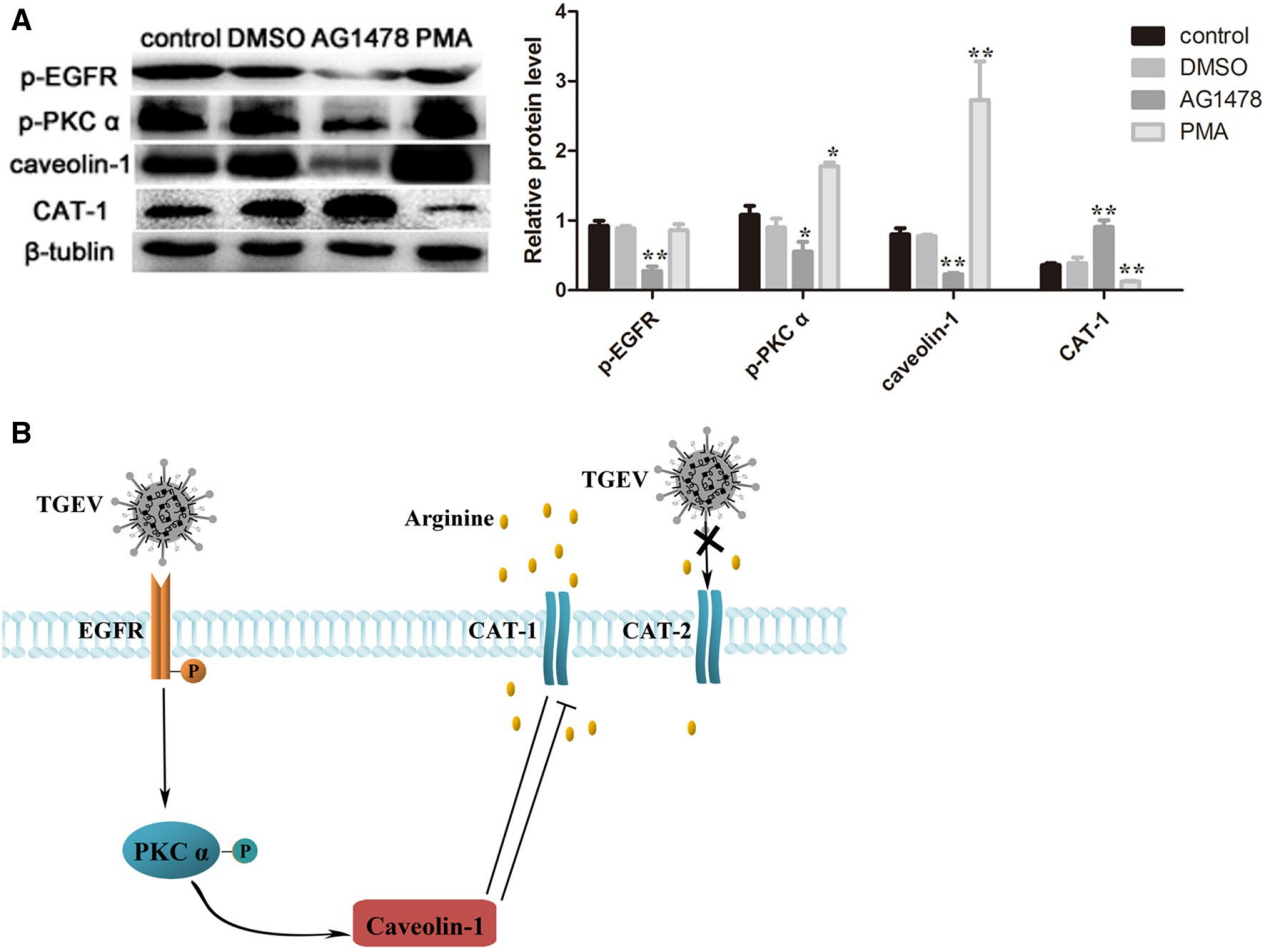
**TGEV infection decreases arginine uptake. A** Cells were treated with PBS, DMSO (100 nM), AG1478 (100 nM) or PMA (100 nM) at 37 °C for 12 h, and Western blot analysis was then used to measure expression of p-EGFR, p-PKC α, caveolin-1 and CAT-1. Differences were considered significant at *0.01 < *p* < 0.05, **p < 0.01. **B** Proposed model showing signaling pathway that regulates arginine uptake during TGEV infection. TGEV infection activates the EGFR-PKC α-caveolin-1 signaling pathway, suppresses CAT-1 expression, and decreases arginine uptake.

Finally, we explored the relationships between EGFR, PKC α, caveolin-1, and CAT-1 to probe their behavior as a signaling pathway. As shown in Figure 6A, treatment of cells with EGFR inhibitor (AG1478) resulted in inhibition of p-EGFR, p-PKC α, and decreased caveolin-1 protein expression, and higher CAT-1 expression. PMA (a PKC α activator) had no influence on p-EGFR, while it increased p-PKC α and caveolin-1, and decreased CAT-1 expression.

## Discussion

Arginine is an essential amino acid in mammals and plays an important role in tissue growth. In the present study, we showed that TGEV infection decreased the uptake of arginine in IPEC-J2 cells. Although four CAT proteins (CAT-1, CAT-2, CAT-3, and CAT-4) mediate arginine transport, only CAT-1 and CAT-2 are expressed in porcine intestinal epithelial cells. It has been demonstrated that CAT-1 is a higher-affinity carrier for cationic amino acids, and has more pronounced trans stimulation compared with CAT-2 [8, 36]. Our experiments showed that expression of CAT-1 was consistent with the trends of arginine uptake, while CAT-2 mRNA levels remained unchanged. These results suggest that CAT-1 may play an important role in mediating arginine uptake during TGEV infection.

Previous studies demonstrated that PKC α acts as a regulator of CAT-1 transporter activity in cells [21]. PKC α activity is controlled by phosphorylation, and activation of PKC α leads to down-regulation of CAT-1 at the cell surface [37]. Treatment with PMA, a PKC stimulant, decreased the uptake of arginine and the expression of CAT-1. Consistent with results reported by Schwartz et al., the modulation of CAT-1 was associated with p-PKC α [38]. We therefore conclude that p-PKC α influences the transport activity of CAT-1.

EGFR belongs to the receptor tyrosine kinase (RTK) family and is activated by many viruses [27, 39]. Activated EGFR triggers numerous downstream signaling pathways, including PKC-mediated cascades, and activates Ras, which affects various MAP kinases [40]. Hu et al. demonstrated that EGFR influences TGEV entry, and plays a synergistic role with APN early in TGEV infection. TGEV acts via the EGFR-PI3 K-Rac1/Cdc42-PAK-LIMK signaling pathway to regulate coflin activity and F-actin arrangement early in infection, and promotes TGEV entry [27]. Further research showed EGFR acts as a co-factor for TGEV entry, and TGEV S1 protein is able to bind to EGFR [28]. Moreover, EGFR is also involve in the regulation of glucose uptake during TGEV infection [41]. Our data provide evidence that EGFR regulates arginine uptake by functioning as a signal molecule in an EGFR-PKC α-caveolin-1-CAT-1 signaling pathway in TGEV-infected cells (Figure 6B). Our model includes a previously unreported signaling pathway that links EGFR with CAT-1. However, it is not yet known how EGFR passes a signal to CAT-1, or if other as yet unidentified signaling molecules regulate CAT-1.

Both PKC and CAT-1 are located in the caveola, suggesting that caveolin-1 may be involved in arginine uptake. Caveolin-1 is the main protein component of caveolae, and is necessary for caveolae formation [42]. CAT-1 and caveolin-1 were reported to co-localize on the surface of cells. Our results showed that increased caveolin-1 levels are involved in the regulation of CAT-1. A possible explanation is that TGEV infection promotes CAT-1 clustering to caveolae islands, resulting in co-location.

In conclusion, we found that TGEV infection induces activation of p-EGFR and p-PKC α, enhances the expression of caveolin-1, attenuates CAT-1 expression, and decreases arginine uptake in IPEC-J2 cells. This study furthers our understanding of the mechanisms of TGEV infection, and is the first description of TGEV infection and amino acid uptake in vitro.

## Abbreviations

TGEV: transmissible gastroenteritis virus
HPLC: high performance liquid chromatography
hpi: hours post-infection
CAT-1: cationic aino acid transporter 1
PKC: protein kinase C
EGFR: epidermal growth factor receptor
IPEC-J2: porcine small intestinal epithelial cell line
DMEM: Dulbecco’s modified Eagle’s medium
FBS: fetal bovine serum
MOI: multiplicity of infection
PBS: phosphate-buffered saline
SDS-PAGE: sulfate polyacrylamide gel electrophoresis
RNAi: RNA interference
PMA: phorbol 12-myristate 13-acetate
